# Supplementary material for: Cancer neoantigen prioritization through sensitive and reliable proteogenomics analysis
Source: Nat Commun. 2020 Apr 9;11:1759. doi: 10.1038/s41467-020-15456-w (PMC7145864; doi:10.1038/s41467-020-15456-w)
Supplement: Supplementary file 1 — Supplementary Information [file 41467_2020_15456_MOESM1_ESM.pdf]

Supplementary information

Cancer neoantigen prioritization through sensitive and reliable proteogenomics analysis

Bo Wen[1,2], Kai Li[1,2], Yun Zhang[1,2], Bing Zhang[1,2#]

[1]Lester and Sue Smith Breast Center, Baylor College of Medicine, Houston, TX 77030, USA

[2]Department of Molecular and Human Genetics, Baylor College of Medicine, Houston, TX 77030, USA

# Correspondence should be addressed to B.Z. (bing.zhang@bcm.edu).

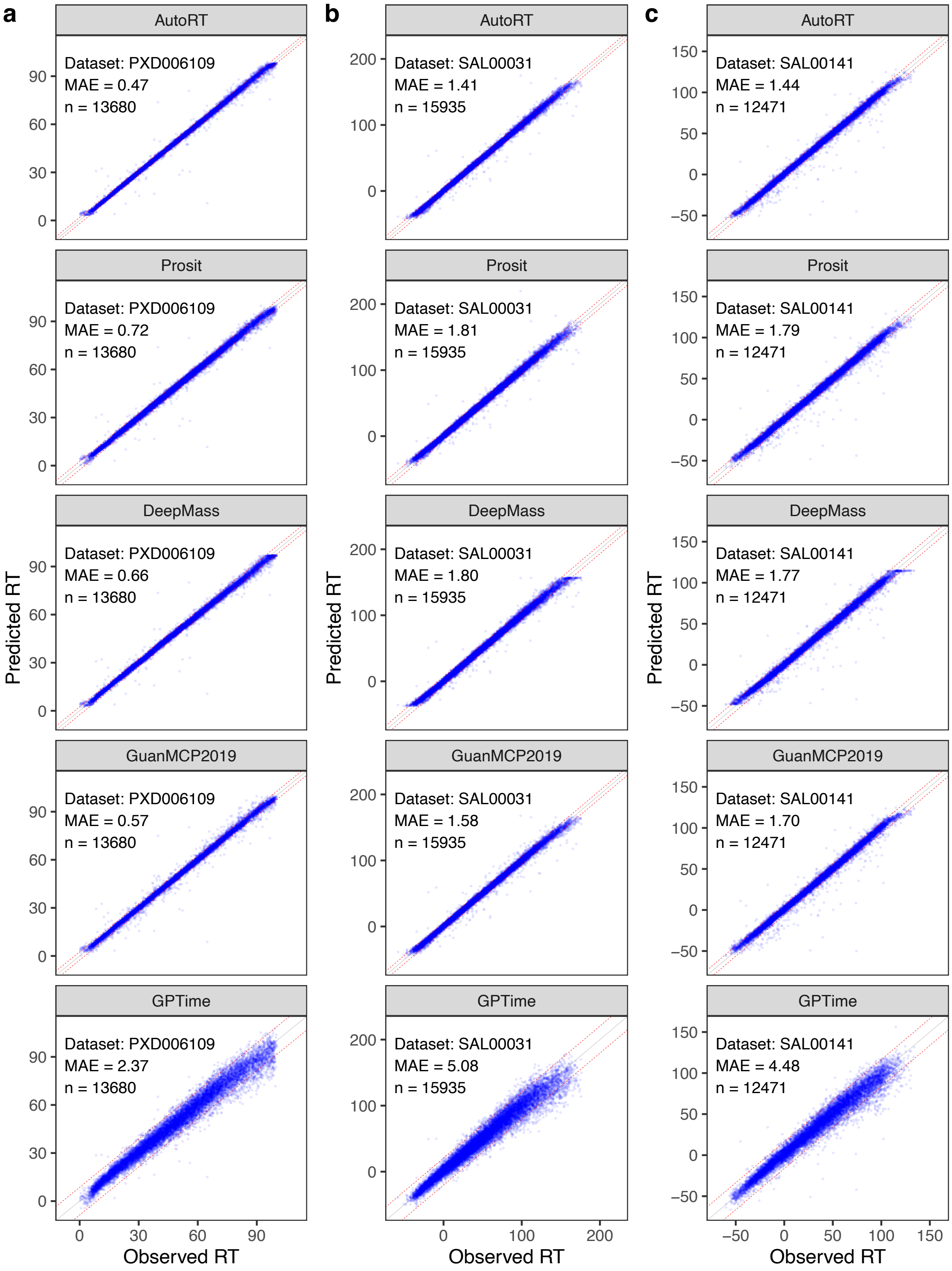

Supplementary Figure 1: Scatter plots of predicted RT against observed RT on dataset (a) PXD006109, (b) SAL00031 and (c) SAL00141. Dashed red lines mark the RT isolation window required to encompass 95% of all peptides around the diagonal (gray solid line).

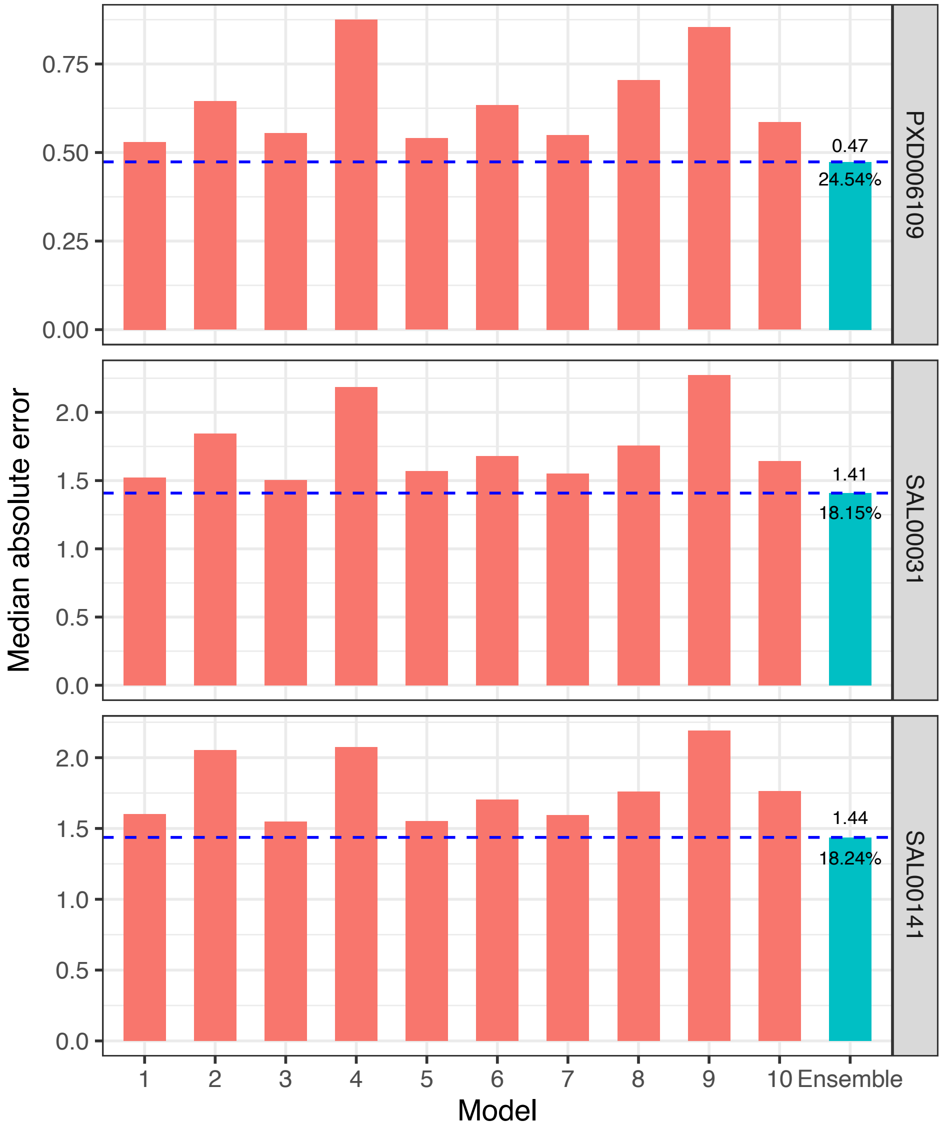

Supplementary Figure 2: Comparison of performance between individual models and the ensemble models. The blue dashed horizontal lines indicate the median absolute errors (MAEs) of the ensemble models. The numbers above the dashed lines are MAEs of the ensemble models and the numbers below the dashed lines are the percentages of performance improvement comparing ensemble models with individual models in terms of MAE.

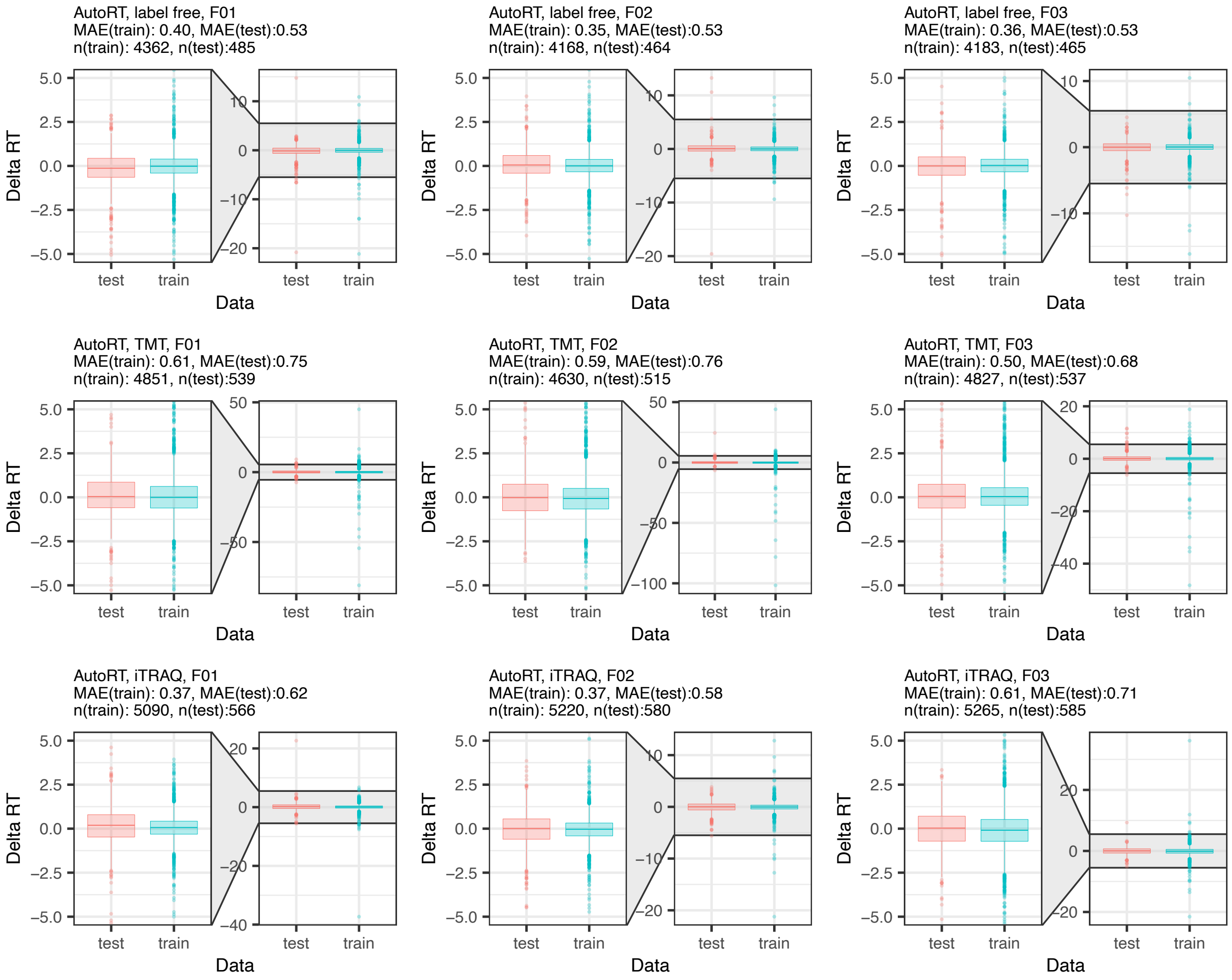

Supplementary Figure 3: Distribution of errors for RT prediction models on training data and testing data from the CPTAC label-free, TMT and iTRAQ datasets. For boxplots, centerline indicates the median, box limits indicate upper and lower quartiles, whiskers indicate the 1.5 interquartile range and points indicate outliers.

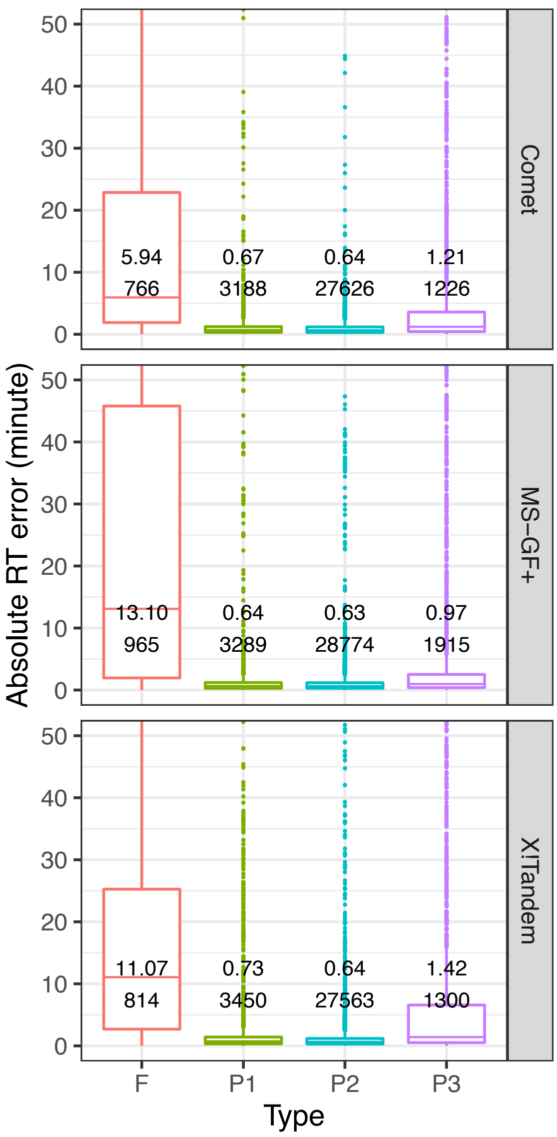

Supplementary Figure 4: Comparison of PepQuery with SpectrumAI. "F": variant peptides failing both PepQuery and SpectrumAI validation. "P1": variant peptides passing only PepQuery validation. "P2": variant peptides passing both PepQuery and SpectrumAI validation. "P3": variant peptides passing only SpectrumAI validation. For boxplots, centerline indicates the median, box limits indicate upper and lower quartiles, whiskers indicate the 1.5 interquartile range and points indicate outliers. For boxplots, the Y axis was limited to up to 50.

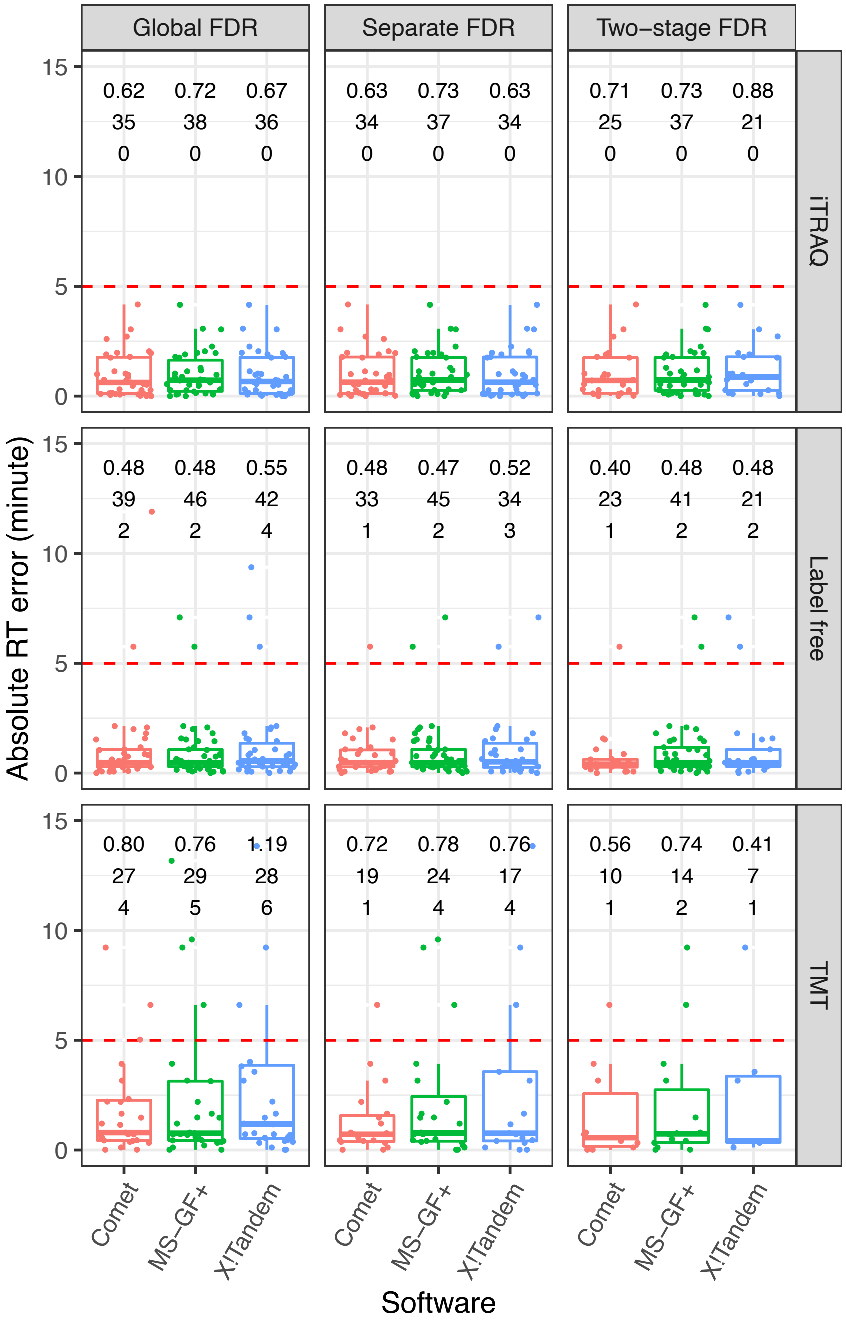

Supplementary Figure 5: Absolute RT error distribution of somatic mutation derived peptides with predicted neoepitopes. The numbers on the first line are the median absolute RT errors. The numbers on the second line are the total identified somatic derived peptides with predicted neoepitopes. The numbers on the third line are the numbers of peptides with absolute RT error greater than 5 minutes. For boxplots, centerline indicates the median, box limits indicate upper and lower quartiles, whiskers indicate the 1.5 interquartile range and points indicate outliers. For boxplots, the Y axis was limited to up to 15.

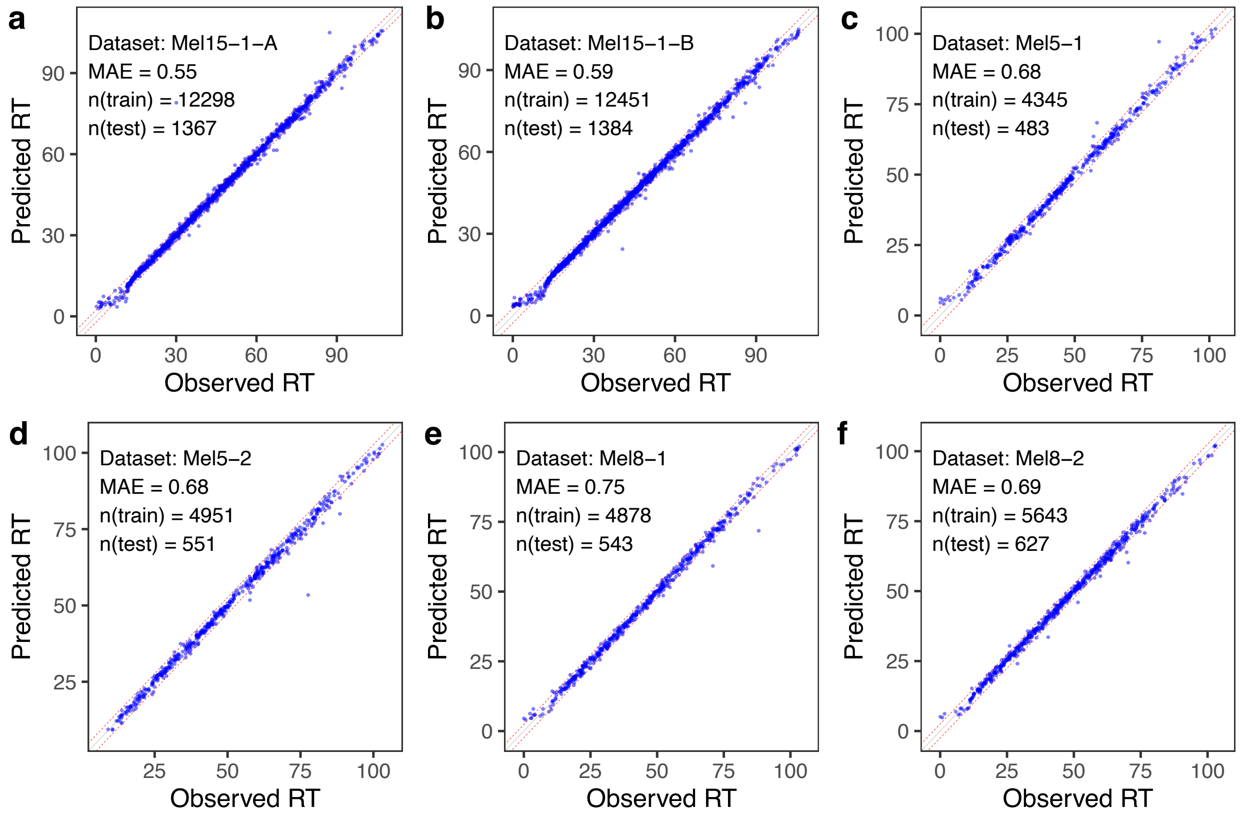

Supplementary Figure 6: Scatter plots of predicted RT against observed RT on the immunopeptidomics data. Dashed red lines mark the RT isolation window required to encompass 95% of all peptides around the diagonal (gray solid line).

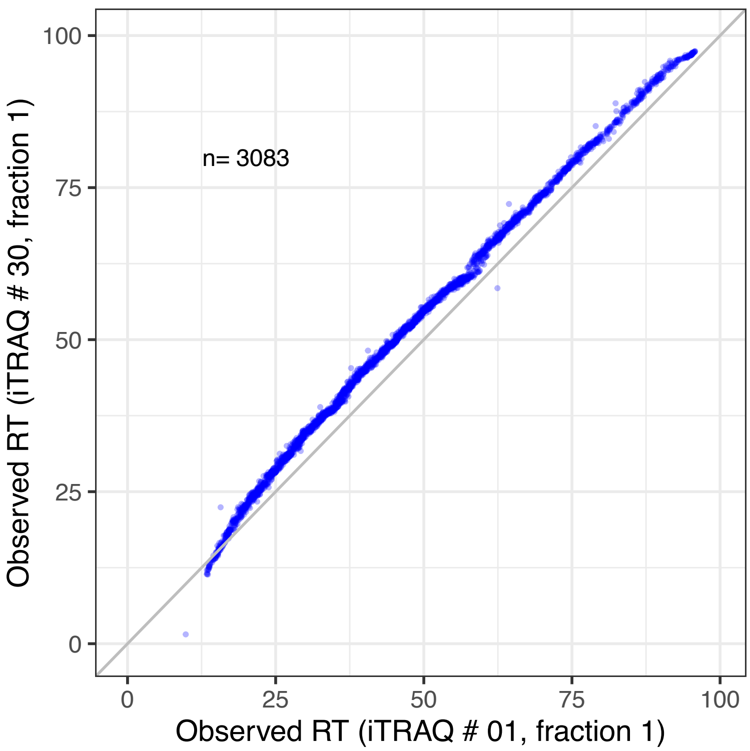

Supplementary Figure 7: Scatter plot of observed RTs for the peptides detected in two different experiments from the same study.

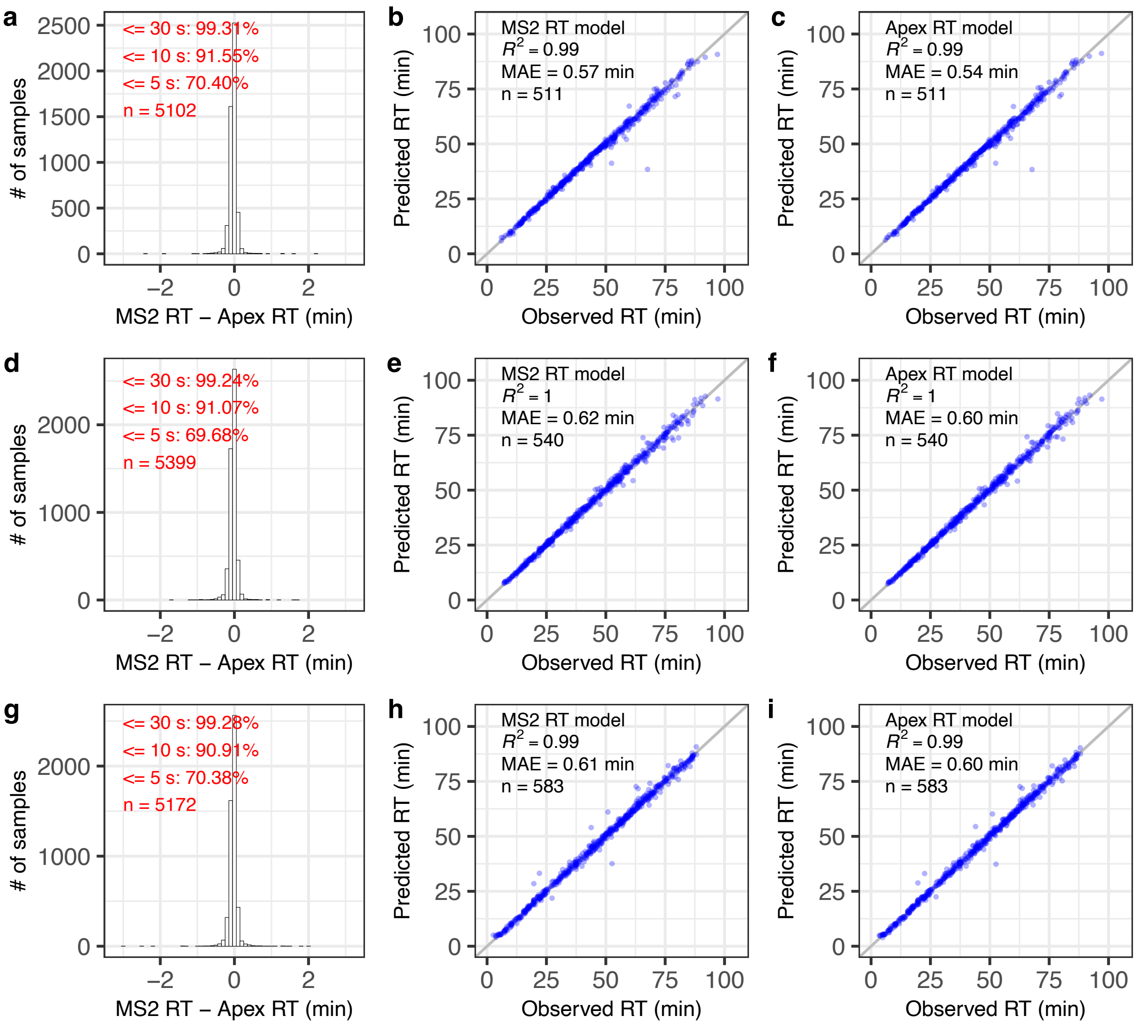

Supplementary Figure 8: Comparison of performance using MS2 RTs with Apex RTs on the label-free data. (a)-(c), (d)-(f) and (g)-(i) represent the comparisons on three fractions, respectively.

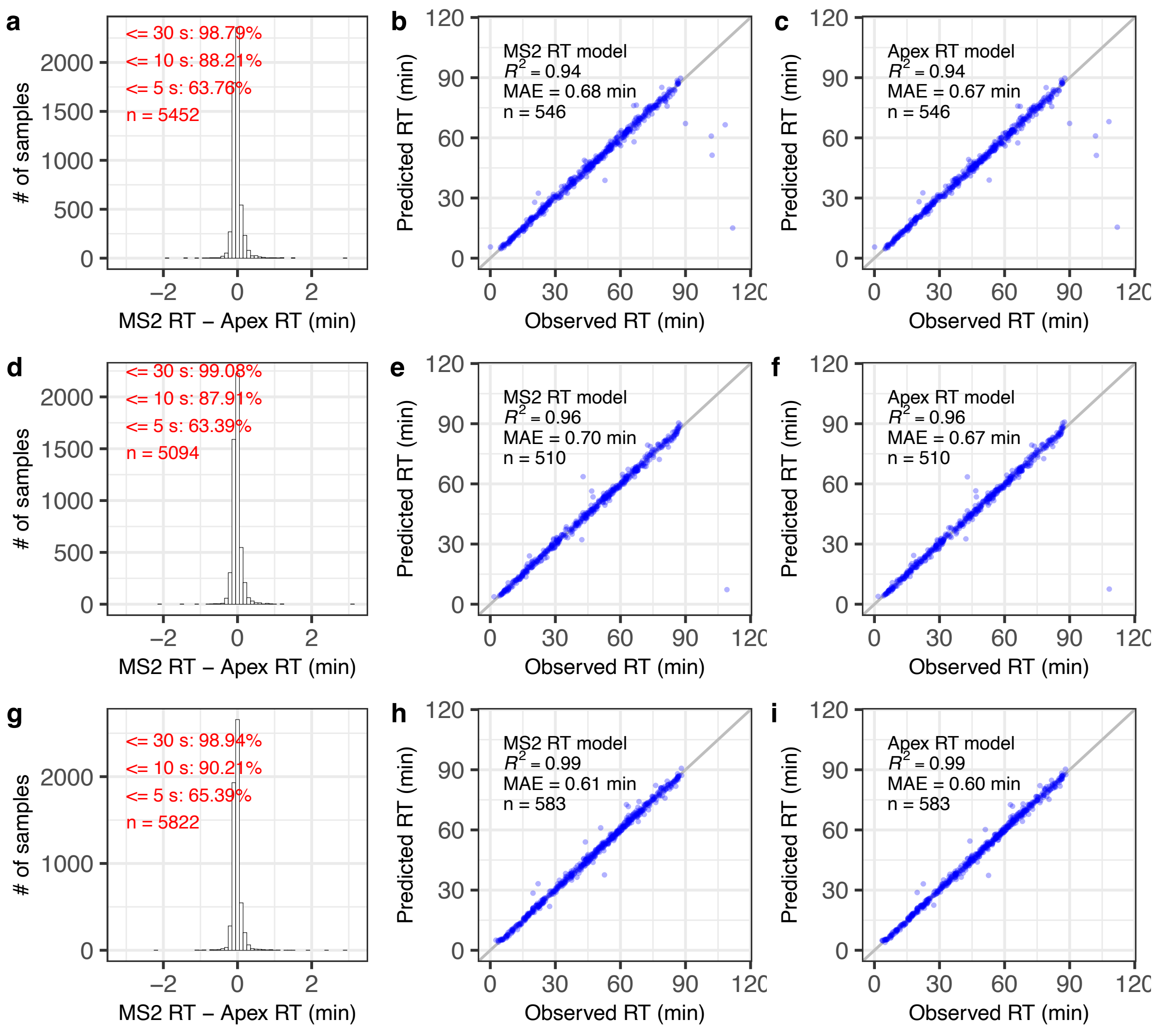

Supplementary Figure 9: Comparison of performance using MS2 RTs with Apex RTs on the TMT data. (a)-(c), (d)-(f) and (g)-(i) represent the comparisons on three fractions, respectively.

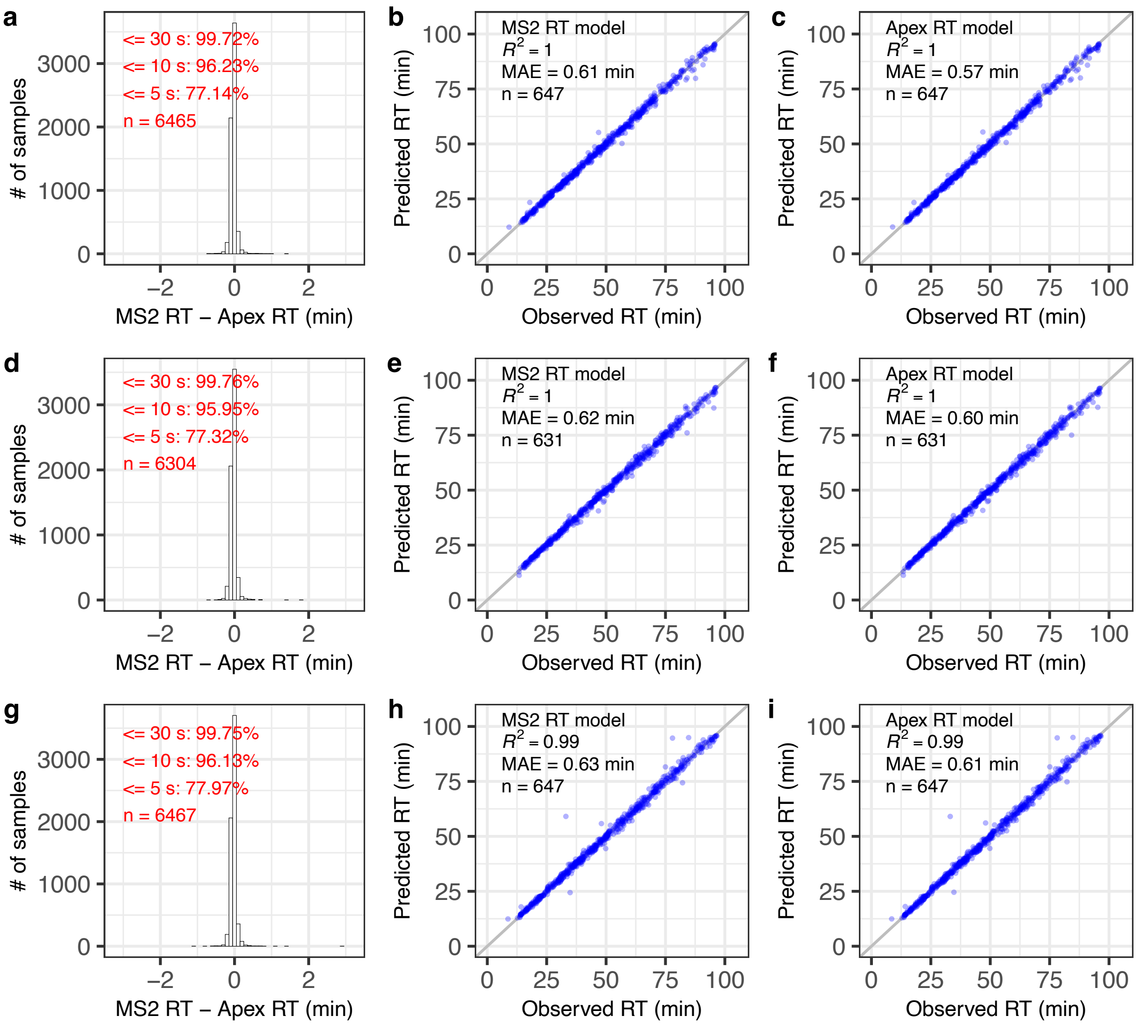

Supplementary Figure 10: Comparison of performance using MS2 RTs with Apex RTs on the iTRAQ data. (a)-(c), (d)-(f) and (g)-(i) represent the comparisons on three fractions, respectively.
