# Supplementary material for: Cancer neoantigen prioritization through sensitive and reliable proteogenomics analysis
Source: Nat Commun. 2020 Apr 9;11:1759. doi: 10.1038/s41467-020-15456-w (PMC7145864; doi:10.1038/s41467-020-15456-w)
Supplement: Supplementary file 3 — Description of Additional Supplementary Files [file 41467_2020_15456_MOESM3_ESM.pdf]

Description of Additional Supplementary Files

File Name: Supplementary Data 1
Description: Variant peptide identification results for the label-free data.

File Name: Supplementary Data 2
Description: Variant peptide identification results for the TMT data.

File Name: Supplementary Data 3
Description: Variant peptide identification results for the iTRAQ data.

File Name: Supplementary Data 4
Description: Somatic variant peptide identification results for the label-free data.

File Name: Supplementary Data 5
Description: Somatic variant peptide identification results for the TMT data.

File Name: Supplementary Data 6
Description: Somatic variant peptide identification results for the iTRAQ data.

File Name: Supplementary Data 7
Description: Neoantigen prediction results for the label-free data.

File Name: Supplementary Data 8
Description: Neoantigen prediction results for the TMT data.

File Name: Supplementary Data 9
Description: Neoantigen prediction results for the iTRAQ data.

File Name: Supplementary Data 10
Description: Somatic variant peptide identification result for the immunopeptidomics data.

File Name: Supplementary Data 11
Description: Genetic algorithm search space for neural architecture search.
